# Differentiation of Club Cells to Alveolar Epithelial Cells *In Vitro*

**DOI:** 10.1038/srep41661

**Published:** 2017-01-27

**Authors:** Dahai Zheng, Boon-Seng Soh, Lu Yin, Guangan Hu, Qingfeng Chen, Hyungwon Choi, Jongyoon Han, Vincent T. K. Chow, Jianzhu Chen

**Affiliations:** 1 Interdisciplinary Research Group in Infectious Diseases, Singapore-Massachusetts Institute of Technology Alliance for Research and Technology, Singapore; 2 A*STAR Institute of Molecular and Cell Biology, 61 Biopolis Drive Proteos, Singapore 138673, Singapore; 3 Department of Biological Sciences, National University of Singapore, 117543, Singapore; 4 Interdisciplinary Research Group in BioSystems and Micromechanics, Singapore-Massachusetts Institute of Technology Alliance for Research and Technology, Singapore; 5 The Koch Institute for Integrative Cancer Research and Department of Biology, Massachusetts Institute of Technology, Cambridge, Massachusetts, United States of America; 6 Saw Swee Hock School of Public Health, National University of Singapore, Singapore; 7 Department of Electrical Engineering and Computer Science, Department of Biological Engineering, Massachusetts Institute of Technology, Cambridge, Massachusetts, United States of America; 8 Host and Pathogen Interactivity Laboratory, Department of Microbiology and Immunology, Yong Loo Lin School of Medicine, National University of Health System, National University of Singapore, Singapore

## Abstract

Club cells are known to function as regional progenitor cells to repair the bronchiolar epithelium in response to lung damage. By lineage tracing in mice, we have shown recently that club cells also give rise to alveolar type 2 cells (AT2s) and alveolar type 1 cells (AT1s) during the repair of the damaged alveolar epithelium. Here, we show that when highly purified, anatomically and phenotypically confirmed club cells are seeded in 3-dimensional culture either in bulk or individually, they proliferate and differentiate into both AT2- and AT1-like cells and form alveolar-like structures. This differentiation was further confirmed by transcriptomic analysis of freshly isolated club cells and their cultured progeny. Freshly isolated club cells express Sca-1 and integrin α6, markers commonly used to characterize lung stem/progenitor cells. Together, current study for the first time isolated highly purified club cells for *in vitro* study and demonstrated club cells’ capacity to differentiate into alveolar epithelial cells at the single-cell level.

The lung is a branching structure of trachea, bronchioles and alveoli, evolved for efficient gas exchange. Under normal conditions the turnover rate of lung cells is low^1^,^2^. In response to injuries, however, lung progenitor cells quickly proliferate and differentiate to repair the damaged structures in order to maintain lung function. Various studies, especially those in mice using cell specific lineage tracing^3^,^4^,^5^,^6^, have identified different cell types in the repair of lung damages^7^,^8^,^9^,^10^. Basal cells, which reside in tracheobronchial epithelium and express transformation related protein 63 (p63) and keratin 5 (Krt5), can self-renew and differentiate into club cells, ciliated cells and goblet cells^3^,^11^,^12^. Club cells, which reside in bronchioles and express secretoglobin family 1A member 1 (Scgb1a1), are progenitors for the repair of bronchiolar epithelium^4^,^13^,^14^,^15^. In alveolar epithelium, alveolar type 2 cells (AT2s), which express pro-surfactant protein C (pro-SPC), are the progenitors of alveolar type 1 cells (AT1s), which express podoplanin (PDPN) and cover more than 90% of the alveolar area^5^,^6^,^16^,^17^. Studies have also characterized and isolated lung stem/progenitor cells using stem/progenitor cell surface markers. Among the reported lung stem/progenitor cell populations are CD31^−^CD45^−^CD34^+^Sca-1^+^ cells^18^, CD45^−^CD31^−^EpCAM^hi^CD49f^+^CD104^+^CD24^low^ cells^19^, and integrin α6β4^+^ (or CD49fCD104^+^) cells^20^, some of which also express CD200 and CD14 and are suggested as lineage negative epithelial progenitor cells (LNEPs)^21^. Despite these progresses, the relationship between stem/progenitor cells identified by lineage tracing and surface staining has yet to be delineated, so as the full differentiation potential of various cell types during the lung damage repair.

We have previously used Scgb1a1-CreER: ACTB-Tm-EGFP transgenic mice to genetically trace club cells during the repair of lung damage induced by influenza virus infection or bleomycin treatment. Our results showed that after severe injuries, club cells were traced to give rise to AT2s and AT1s to regenerate alveolar epithelia^22^,^23^ and the p63^+^ basal-like cells in damaged lung parenchyma to generate new bronchioles^24^. These results are consistent with other reports showing that the newly generated AT2s are not derived from existing AT2s during lung damage repair^20^. Yet, it has not been possible to show if a single club cell can give rise to both AT1 and AT2 by lineage tracing in mice. In the present study, we have addressed this question by differentiating highly purified club cells, either in bulk or individually, into both AT2- and AT1-like cells in 3-dimensional (3-D) culture. Our quantitative and transcriptomic analyses provide further evidence for club cell to AT2 and AT1 cell differentiation.

## Results

### Club cells form colonies in 3-D culture

To study the differentiation potential of club cells, we employed a 3-D culture *in vitro* using purified club cells^25^. As there is no known unique surface markers for live club cells sorting by flow cytometry, we took advantage of Scgb1a1-CreER: ACTB-Tm-EGFP transgenic mice where club cells are positive for enhanced green fluorescent protein (EGFP)^22^,^23^. In this transgenic system, CreER is expressed in Scgb1a1^+^ club cells but retained in the cytoplasm. Upon TMX treatment CreER is translocated to the nucleus where it catalyzes recombination to delete the tomato red transgene and turn on EGFP expression. Theoretically, in the absence of TMX treatment, all transgenic cells, including Scgb1a1^+^ club cells, express tomato red^26^. Upon TMX treatment, club cells lose tomato red expression and become EGFP positive. However, ~10% of club cells in the bronchioles are EGFP^+^ in the absence of TMX treatment^4^,^22^,^23^.

**Figure 1:**
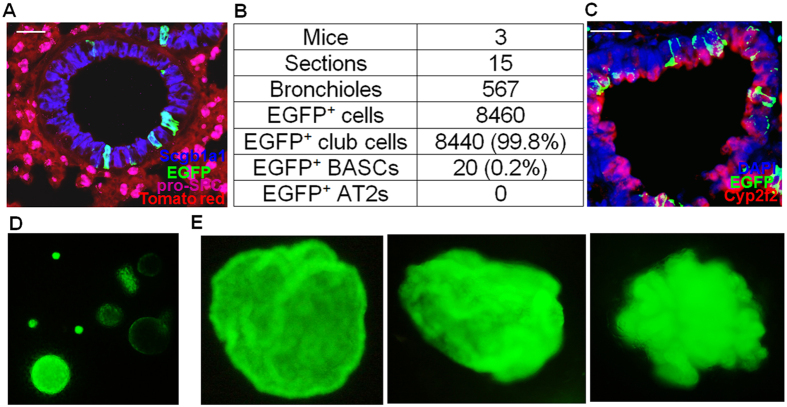
Club cells form colonies in 3-D culture. (**A**) Representative Scgb1a1 (blue), EGFP (green), pro-SPC (magenta) and tomato red (red) staining of lung sections of scgb1a1-CreER: ACTB-Tm-EGFP transgenic mice without tamoxifen treatment. (**B**) Quantification of stained cells in distal lung sections. The number of mice, lung sections, bronchioles, EGFP^+^ cells, EGFP^+^ club cells (Scgb1a1^+^ pro-SPC^-^), EGFP^+^ BASCs (Scgb1a1^+^ pro-SPC^+^), and EGFP^+^ AT2s (Scgb1a1^−^ pro-SPC^+^) are indicated. (**C**) Representative DAPI (blue), EGFP (green) and Cyp2f2 (red) staining of lung sections of scgb1a1-CreER: ACTB-Tm-EGFP transgenic mice without tamoxifen treatment. (**D,E**) Representative photographs of EGFP^+^ colonies in 3-D culture of EGFP^+^ club cells for 6 (**D**) and 10 (**E**) days. Scale bars: (**A,C**) 25 μm.

We further determined the identity of EGFP^+^ cells in Scgb1a1-CreER: ACTB-Tm-EGFP transgenic mice without TMX treatment. To reduce the number of EGFP^+^ ciliated cells derived from EGFP^+^ club cells, 6-week-old mice were used in our experiment. Lung sections were stained for Scgb1a1 and pro-SPC. Among 8460 individual EGFP^+^ cells examined in 15 distal lung sections from 3 transgenic mice, 8440 (99.8%) were Scgb1a1^+^ but pro-SPC^−^ and localized in bronchiolar epithelia (Fig. 1A and B), suggesting they are club cells. Consistently, most of the EGFP^+^ club cells were also positive for cytochrome P450, family 2, subfamily f, polypeptide 2 (Cyp2f2) (Fig. 1C), another marker for club cells^27^. Only 20 of the 8460 EGFP^+^ cells (0.2%) were weakly positive for both Scgb1a1 and pro-SPC and resided at bronchoalveolar duct junctions (BADJs), suggesting they might be the reported bronchioalveolar stem cells (BASCs)^18^. Thus, without TMX treatment 99.8% of EGFP^+^ cells in the distal lung in Scgb1a1-CreER: ACTB-Tm-EGFP transgenic mice are the typical club cells, which are negative for pro-SPC.

EGFP^+^ cells from transgenic mice without TMX treatment (around 0.1–0.2% of the total lung cells) were purified by cell sorting (~95% purity) and placed in 3-D culture as described^25^. EGFP^+^ colonies became visible under fluorescent microscope 48 hours after seeding. The colonies increased in size with time and were spherical before day 6, but gradually collapsed and became irregular in shape afterwards (Fig. 1D and E). The size of the colonies varied significantly: some were already quite large by day 6, whereas others were still quite small. By plating 250, 500, and 1000 of EGFP^+^ cells per well and counting the numbers of colonies at day 10, the colony forming efficiency (CFE) of the EGFP^+^ cells was 10.47 ± 1.03% (mean ± SD). Very rarely did we find tomato red positive colonies which were likely formed by contaminating EGFP^−^ cells. Since only EGFP^+^ colonies were counted, such contamination should not contribute to the quantification. Because EGFP^+^ BASCs only constituted ~0.2% of the total EGFP^+^ cells, even if all of them formed colonies in the 3-D culture, still most (~ 98%) of the colonies should be derived from typical club cells. Thus, club cells are capable of forming colonies *in vitro*, indicating their strong proliferation capacity.

EGFP^+^ cells were also isolated from TMX treated Scgb1a1-CreER: ACTB-Tm-EGFP mice where the percentage of EGFP^+^ cells was ~2% of the total lung cells. Following 3-D culture, EGFP^+^ cells also formed colonies but CFE was 5.63 ± 1.45%. Due to the presence of EGFP^+^ AT2 cells in the TMX-treated mice^22^,^23^, we did not study these colonies in details.

In addition, EGFP^+^ cells were purified from TMX-treated and non-treated Scgb1a1-CreER: ACTB-Tm-EGFP mice and further fractionated into two groups based on cell size using inertial microfluidic separation technique^28^,^29^,^30^. Following 3-D culture, both the large and small EGFP^+^ cells formed colonies. The large EGFP^+^ cells from non-treated mice had a CFE of 14.4 ± 2.00%, while the small EGFP^+^ cells from non-treated mice had a CFE of 6.1 ± 0.76%. In comparison, the large EGFP^+^ cells from TMX-treated mice had a CFE of 6.5 ± 1.44%, and the small EGFP^+^ cells from TMX-treated mice had a CFE of 3.0 ± 1.23%. This data indicates that the large club cells are more efficient in forming colonies *in vitro*.

### Club cells differentiate into alveolar cells in 3-D culture

**Figure 2:**
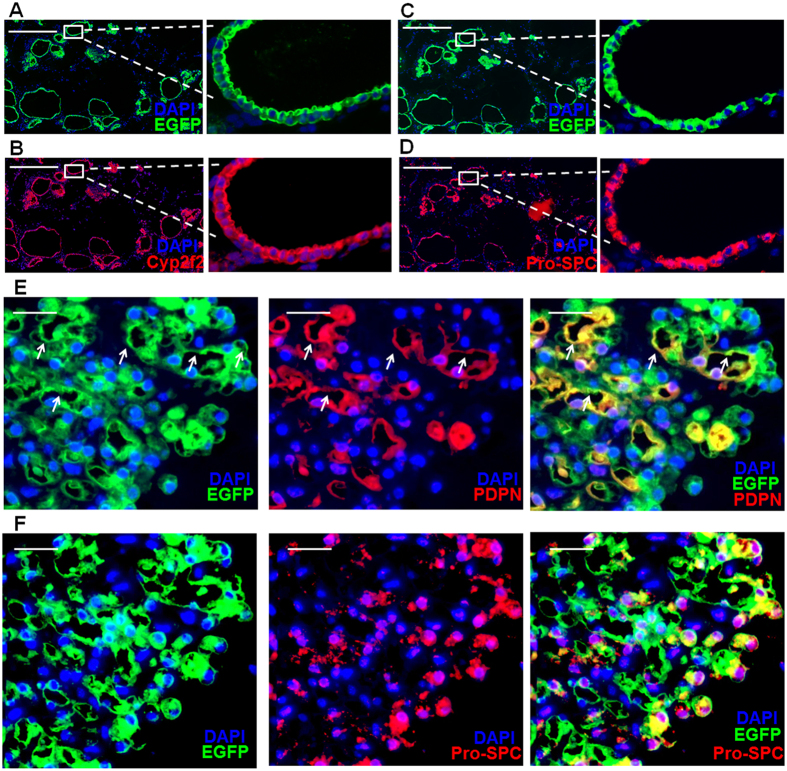
Club cells differentiate into alveolar cells in 3-D culture. (**A,B**) Representative DAPI (blue), EGFP (green) and Cyp2f2 (red) staining of colonies formed by EGFP^+^ club cells at day 7. Channels of EGFP and Cyp2f2 are shown separately in panel (A) and (B) for clarity. Higher magnification of the same selected area in (**A**) and (**B**) are shown as indicated. (**C,D**) Adjacent section of (**A**) is stained for DAPI (blue), EGFP (green) and pro-SPC (red). Channels of EGFP and pro-SPC are shown separately in panel (C) and (D). Higher magnification of the same selected area in (**C**) and (**D**) are shown as indicated. (**E**) Representative DAPI (blue), EGFP (green) and PDPN (red) staining of colonies formed by EGFP^+^ club cells at day 14. Combinations of different channels are shown as indicated in different panels. Arrows indicate cells positive for both EGFP and PDPN in alveolar-like structures. (**F**) Adjacent section of (**E**) is stained for DAPI (blue), EGFP (green) and pro-SPC (red). Combinations of different channels are shown as indicated. Scale bars: (**A–D**) 500 μm; (**E,F**) 25 μm.

To determine club cell differentiation in 3-D culture, we processed the colonies for paraffin sectioning and stained the sections for markers of club cells, AT1s and AT2s. By day 7, expression of Scgb1a1 was undetectable from the EGFP^+^ colonies (data not shown), even though all the cells were Scgb1a^+^ at the start of the culture. In all EGFP^+^ colonies, the majority of the cells remained positive for Cyp2f2 (Fig. 2A and B) and the majority of the cells in the adjacent section were also positive for pro-SPC (Fig. 2C and D). Consistent results were obtained in three independent experiments where 308 EGFP^+^ colonies from 6 mice were analyzed. Only a few colonies (around 3% of the total colonies) contained EGFP^+^ p63^+^ cells (Supp Fig. S1). Therefore, most of the cells in the EGFP^+^ colonies by day 7 of culture are Cyp2f2 and pro-SPC double positive, resembling the pro-SPC^+^ bronchiolar epithelial cells (SBECs) *in vivo*^22^. This is also consistent with the previous observation that after isolation, club cells switch on the expression of pro-SPC^31^.

By day 7, although the colonies varied in shape, sections of colonies still had a monolayer of cells encompassing a luminal space. By day 14, the size of colonies became much bigger and the structures were also more complex. Majority of the colonies (~78%) contained alveolar-like structures, with PDPN^+^ AT1-like cells and pro-SPC^+^ AT2-like cells (Fig. 2E and F). In contrast, Scgb1a1 and Cyp2f2 expressions were lost based on analysis of a total of 112 colonies in three independent experiments (data not shown). Since 99.8% of the EGFP^+^ cells are initially typical club cells, these results clearly show that club cells can differentiate into AT2- and AT1-like cells *in vitro*.

### A single club cell can differentiate into alveolar cells in 3-D culture

**Figure 3:**
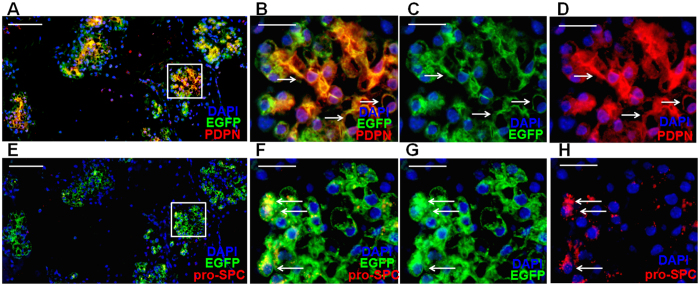
A single club cell can differentiate into alveolar cells in 3-D culture. (**A–D**) Representative DAPI (blue), EGFP (green) and PDPN (red) staining of secondary colonies derived from a single EGFP^+^ club cell. Higher magnification of selected area in A with combination of different channels was shown as B-D. Arrows indicate cells positive for both EGFP and PDPN in alveolar-like structures. (**E–H**) Adjacent section of (**A**) was stained for DAPI (blue), EGFP (green) and pro-SPC (red). Higher magnification of selected area in E with combination of different channels was shown as **F–H**. Arrows indicate cells positive for both EGFP and pro-SPC in alveolar-like structures. Scale bars: (**A,E**) 100 μm; (**B–D**, **F–H**) 25 μm.

To study the proliferation and differentiation potential of individual club cells, we sorted EGFP^+^ club cells from transgenic mice without TMX-treatment, counted, diluted and plated in 96-well plate in such a dilution that each well received on average of 0.8 cell. The actual number of cells in each well was visually assessed by fluorescent microscopy and those wells that had only one cell were marked. The single cells from the marked wells were processed individually for 3-D culture. Out of 384 single cells processed for culture, 26 colonies were obtained by day 9 (a CFE of ~7%). The primary colonies were dissociated and cells were re-seeded into new wells and cultured. Another 9 days later, the secondary colonies were processed for sectioning and staining. Out of 427 secondary colonies examined, none of them contained cells positive for Scgb1a1, Cyp2f2 or p63 (data not shown), but 72% of the colonies had alveolar-like structures with both PDPN^+^ AT1-like cells and pro-SPC^+^ AT2-like cells (Fig. 3). Thus, as in bulk culture, a single club cell can proliferate and differentiate into AT1- and AT2-like cells in 3-D culture.

### EGFP^+^ club cells express progenitor cell markers Sca-1 and integrin α6

**Figure 4:**
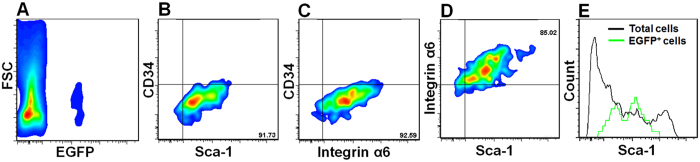
EGFP^+^ club cells are positive for progenitor cell markers. (**A**) Forward scatter (FSC) vs. EGFP plot of total lung cells. (**B–D**) CD34 vs. Sca-1, CD34 vs. integrin α6, and integrin α6 vs. Sca-1 staining profiles of EGFP^+^ cells. (**E**) Histogram showing Sca-1 staining intensities of EGFP^+^ club cells (green trace) and total lung cells (black trace).

To characterize EGFP^+^ club cells in Scgb1a1-CreER: ACTB-Tm-EGFP transgenic mice without TMX treatment, we prepared single cell suspension of distal lung and stained cells for progenitor cell markers, including CD34, Sca-1 and integrin α6. Majority (>85%) of the EGFP^+^ club cells were negative for CD34 but positive for both Sca-1 and integrin α6 (Fig. 4A–D). By comparing Sca-1 expression on total lung cells and EGFP^+^ cells, most of the EGFP^+^ club cells were Sca-1^low^ (Fig. 4E). This result is consistent with previous reports showing club cells express Sca-1^31^, ~30% of CD45^−^CD31^−^EpCAM^hi^CD49f^+^CD104^+^CD24^low^ lung progenitor cells are club cells^19^ and ~10% of integrin α6β4^+^ (or CD49fCD104^+^) lung progenitor cells are strongly positive for Scgb1a1^20^,^21^, and consistent with club cells’ progenitor property *in vitro* and *in vivo*^22^,^23^,^24^.

### Transcriptional profile of EGFP^+^ cells during 3-D culture

To further characterize EGFP^+^ club cells and molecular changes during their *in vitro* culture, we performed RNAseq of freshly purified EGFP^+^ club cells (D0) and purified EGFP^+^ cells from primary colonies at day 4 (D4) and 7 (D7) of culture (Supp Table 1). Each RNA sample yielded ~40 million reads and only 0.36–3.9% of sequences were from ribosomal RNA, indicating high quality of sequencing. Between D4 and D0, D7 and D0, and D7 and D4, 577, 182 and 363 genes were differentially expressed, respectively (Supp Fig. S2 and Supp Table 2). Functional enrichment analysis of differentially expressed genes showed that many of these genes were involved in cell cycle and mitosis, cell development and differentiation, transcription and translation (Supp Table 3), consistent with proliferation and differentiation of club cells during the 3-D culture.

**Figure 5:**
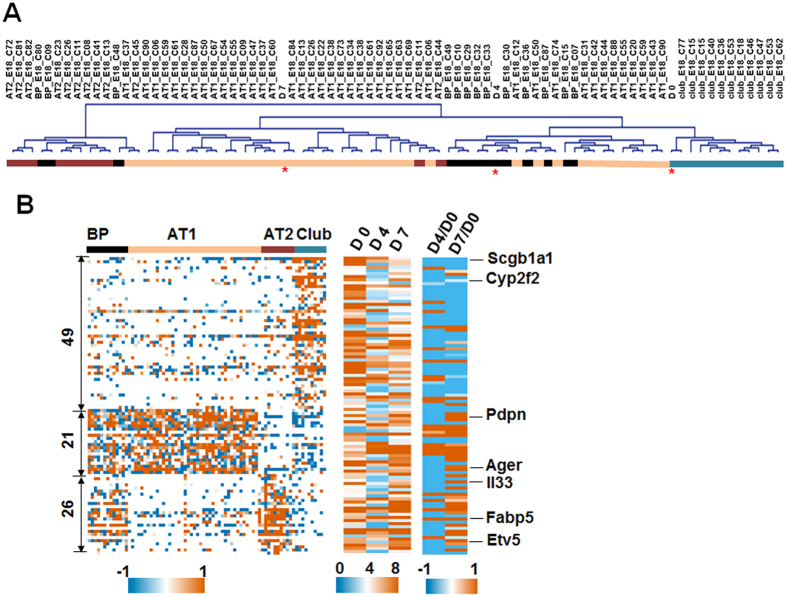
Transcriptional analysis of EGFP^+^ club cells before and after culture. (**A**) RNA of EGFP^+^ cells that were freshly isolated from mouse lung at day 0 (D0), or isolated from the cultured colonies at day 4 (D4) and day 7 (D7) were processed for RNA sequencing. Expression profiles were normalized by mean to zero across all samples for each gene. The expression profiles of published 77 fetal lung single cells (including bipotential progenitor cells (BP), AT1s, AT2s and club cells) were also normalized with the same method. Total matched genes between two dataset were hierarchically clustered using uncentered Pearson method in MeV. Positions of D0, D4 and D7 were indicated with *. (**B**) The group information is the same as in (**A**). A total of 96 differentially expressed genes were matched to the signature genes identified previously by Principle Component Analysis (PCA) of fetal lung cell types (left panel). Expression levels (Log2 CMP) of these genes in D0, D4 and D7 are shown in the middle panel. Fold changes (Log2) of these genes in D4 and D7 against D0 are shown in the right panel with some signature genes labeled.

Transcriptional profiles of mouse fetal lung cell types including 13 bipotential progenitor cells (BP), 42 AT1s, 11 AT2s and 11 club cells were reported recently^32^. To compare EGFP^+^ cell samples (D0, D4 and D7) and the published fetal lung cells, we normalized two data sets with mean to zero at a comparable level and group them based on the expression. A total of 8341 expressed genes were identified from both data sets. Hierarchical clustering showed that the transcriptional profile of freshly isolated EGFP^+^ club cells (D0) was very similar to that of fetal lung club cells, and *in vitro* differentiated cells at Day 4 and Day 7 were most similar to BPs and AT1/AT2s, respectively (Fig. 5A), indicating that club cells may first acquire a progenitor cell status and then differentiate into alveolar cell types. From the differentially expressed genes among D0, D4 and D7, we identified 49, 26 and 21 genes that matched the published signature genes of fetal lung club cells, AT2s and AT1s, respectively (Supp Table 4). Among these 96 genes, the down-regulated ones during the 3-D culture were enriched with club cell signature genes, such as Scgb1a1 and Cyp2f2, while genes up-regulated during the culture were enriched with AT2s and AT1s signature genes, such as Etv5 and Pdpn (Fig. 5B). These data provide further molecular evidence for the differentiation of EGFP^+^ club cells towards AT2s and AT1s in 3-D culture.

## Discussion

Club cells have been well accepted as the regional restricted adult progenitor cells for the repair of bronchiolar epithelium. By lineage tracing in Scgb1a1-CreER: ACTB-Tm-EGFP transgenic mice we have shown previously that club cells can also give rise to AT2s and AT1s during the repair of severe lung damage following either influenza virus infection or bleomycin treatment^23^. We further show that club cell to AT2 and AT1 differentiation goes through a process involving an intermediate stage of pro-SPC^+^ bronchiolar epithelial cells (SBECs) similar as in the fetal lung development^22^. One caveat of our previous study is that TMX treatment of Scgb1a1-CreER:ACTB-Tm-EGFP transgenic mice also induces expression of EGFP in BASCs and a small fraction of AT2s, raising the possibility that the EGFP-labeled AT2s and AT1s are derived from pre-existing AT2s. Although this is unlikely because genetic lineage tracing studies have provided direct evidence that majority of the newly generated AT2s during lung damage repair are not derived from pre-existing pro-SPC^+^ cells including AT2s and BASCs^20^, the issue has persisted. In the present study, we have excluded the complication of EGFP^+^ AT2s by not using TMX treatment of Scgb1a1-CreER: ACTB-Tm-EGFP transgenic mice. Among 8460 EGFP^+^ cells examined, none of them were AT2s, suggesting a frequency of less than 0.01%. EGFP expression by club cells in Scgb1a1-CreER: ACTB-Tm-EGFP transgenic mice enabled us to purify these cells by cell sorting. By *in vitro* differentiation of the highly purified EGFP^+^ cells either in bulk culture or single cell culture, we show that EGFP^+^ club cells are capable of proliferation and differentiation into pro-SPC^+^ AT2-like cells and PDPN^+^ AT1-like cells in apparently alveolar structures. As *in vivo*, the differentiation process also goes through an intermediate pro-SPC^+^ stage. Since only 0.2% of EGFP^+^ club cells reside at the bronchoalveloar duct junction and therefore may be BASCs, even if each and every BASC forms colony in the 3-D culture, their contribution is ~2%, leaving ~98% of the colonies being derived from typical club cells. Thus, results from the *in vitro* differentiation, especially from single club cells, presented here and our previous *in vivo* lineage tracing in Scgb1a1-CreER: ACTB-Tm-EGFP transgenic mice provide unequivocal evidence for the differentiation of club cells to AT2s and AT1s.

It is notable that club cell proliferation and differentiation *in vivo* occur following lung tissue damage, such as that induced by influenza virus infection or bleomycin treatment. In contrast, club cells readily undergo proliferation and differentiation *in vitro* following isolation from healthy mice without influenza virus infection or bleomycin treatment. This difference is consistent with the low turnover rate of lung epithelial cells under physiological conditions and suggests that influenza virus or bleomycin does not play any special role beyond causing tissue damage *in vivo*. Furthermore, this data also suggests that club cells are normally inhibited from proliferation and differentiation *in vivo*. Once the inhibition is released, such as following lung tissue damage or following isolation, they undergo proliferation and differentiation.

We show that the anatomically confirmed EGFP^+^ club cells express integrin α6 and Sca-1, markers commonly used to characterize and identify lung stem/progenitor cells. Our result is consistent with a previous report showing expression of Sca-1 and pro-SPC by a large fraction of club cells^31^. These results raise the possibility that club cells may have contributed to the previously reported lung stem/progenitor cell populations. BASCs are supposedly Scgb1a1^+^ and pro-SPC^+^ and reside at BADJs, however, studies with purified BASCs were actually carried out using CD31^−^CD45^−^CD34^+^ Sca-1^+^ cells^18^. Similarly, ~30% of CD45^−^CD31^−^EpCAM^hi^CD49f^+^ CD104^+^ CD24^low^ lung progenitor cells are club cells^19^ and ~10% of integrin α6β4^+^ (or CD49fCD104^+^) lung progenitor cells are strongly positive for Scgb1a1^20^. Recently, the CD200^+^ CD14^+^ subpopulation of the integrin α6β4^+^ cells was suggested to be the lineage negative epithelial progenitor cells^21^. CD14 is the co-receptor of toll like receptor 4 (TLR4) and club cells are known to be TLR4^+^ and very sensitive to LPS stimulation^33^. Therefore, lung stem/progenitor cells previously isolated using a combination of integrin α6β4, Sca-1, and other markers may have included club cells. In our study, we used EGFP to isolate anatomically confirmed club cells with high purity, which exclude the possible contamination by BASCs (Scgb1a1^+^ pro-SPC^+^ cells) or lineage negative cells.

Repair of alveolar damage by club cells are consistent with fetal lung development and for efficient restoration of lung function. The primary function of the lung is for efficient gas exchange, which requires unimpeded airflow from proximal to distal lung. Following lung injuries due to infection or exposure to chemicals, the relatively fragile parts of the lung, such as alveoli and small bronchioles, are easily damaged. To rebuild a branching structure for efficient airflow, it would appear more difficult to first generate alveoli and hierarchic small bronchioles in the distal airways and then accurately connect them to the proximal airways. In contrast, rebuilding along proximal to distal direction as during fetal lung development would be easier to rebuild a branching structure for unimpeded airflow. Our results showing club cell to AT2 and AT1 differentiation are consistent with this notion.

In conclusion, our quantitative analysis of *in vitro* differentiation and transcriptional analysis of purified club cells and their progeny provide further evidence for club cell to alveolar cell differentiation.

## Methods

### Mice

Transgenic ACTB-mT-EGFP (stock number 007676) mice on the B6 background were purchased from the Jackson Laboratories. Scgb1a1-CreER transgenic mice were kindly provided by Dr. Brigid Hogan of Duke University^4^. Scgb1a1-CreER:ACTB-mT-EGFP double transgenic mice were generated by breeding Scgb1a1-CreER mice with ACTB-mT-EGFP mice in the animal facility at the National University of Singapore. Mice at 6 weeks of age were used for experimentation. All animals were housed in biosafety level 2 animal facilities at NUS.

### Ethics statement

This study was carried out in strict accordance with the National Advisory Committee for Laboratory Animal Research (NACLAR) Guidelines in facilities licensed by the Agri-Food and Veterinary Authority of Singapore (AVA). The protocol with the number 123/11 was approved by the Institutional Animal Care and Use Committee (IACUC) of National University of Singapore.

### Single cell preparation, isolation and *in vitro* culture

Mice were anesthetized with Ketamine/meditomidine, the lungs were perfused with 10 mL PBS through the right ventricle. Trachea and bronchia were removed and only the distal lung was collected, cut into small pieces and digested using collagenase type I (3 mg/ml in PBS, 2 ml per lung) for 1 hour at 37° C. The suspension was further dissociated through an 18 gauge needle and centrifuged at 400 g for 5 minutes. Red blood cells were lysed using 3 ml of ACK lysing buffer (Thermal Fisher Scientific) for 2 minutes, diluted using 20 ml of PBS, and centrifuged at 400 g for 5 minutes. Cells were re-suspended with PBS containing 3% fetal calf serum (FCS), filtered through a 40 μm cell strainer (BD Biosciences) to obtain the single cell suspension. EGFP^+^ cells were sorted using cell sorter FACSAria II (BD Biosciences), and collected in culture medium. Antibodies used for analysis of cell surface markers include CD34-APC, Sca-1-PE/Cy7, integrin α6-PE/Cy7 and integrin α6-APC (all from BioLegend).

Purified EGFP^+^ Cells were cultured under 3-D culture conditions in 24-well transwell with MLg cells as feeder cells. The culture medium and culture conditions are the same as previously described^25^. At day 7 and 14, the samples were fixed using 10% neutral buffered formalin (Sigma) for 15 minutes, embedded with 3% agarose, processed for paraffin embedding and sectioned at 3 μm thickness.

To culture single cell colonies, the isolated EGFP^+^ cells are diluted and aliquoted into 96-well plate. The presence of a single cell in a well was confirmed visually using fluorescent microscope. The single cell was then seeded as described above. The single colony at day 9 was released from matrigel using cell recover solution (BD Biosciences) at 4° C, the colony was then dissociated using collagenase/dispase (Roche Life Science) (2 mg/ml) for 30 minutes at 37° C. The cells were washed and collected by centrifuging at 400 g for 3 minutes and seeded again for 3-D culture to obtain secondary colonies.

### Immunofluorescence staining

Paraffin sections were de-waxed and rehydrated. Antigen retrieval was carried out by microwaving the slides in 0.01 M sodium citric acid buffer (pH 6.0) for 30 min. Sections were then immersed for 1 hour in blocking buffer (3% BSA, 0.2% Triton X-100 in PBS), then incubated in primary antibody (in blocking buffer) at 4° C overnight, followed by incubation with secondary antibody at 4° C for 1 hour. Slides were mounted with antifade reagent with or without DAPI (Life Technologies), and then scanned with a high-resolution MIRAX MIDI system (Carl Zeiss) equipped with both bright field and fluorescence illumination. Images were analyzed by the MIRAX Viewer software.

Polyclonal rabbit anti-Scgb1a1 antibody (US Biological, C5828) was used at 1:200 dilution. Goat anti-pro-SPC (Santa Cruz Biotechnology, sc-7706), rabbit anti-Cyp2f2 (Santa Cruz Biotechnology, sc-67283), goat anti-PDPN (R&D Systems, AF3244), monoclonal mouse anti-p63 4A4 (Santa Cruz Biotechnology, sc-8431), rabbit anti-GFP (Abcam, ab290), goat anti-GFP(Abcam, ab5450), and mouse anti-GFP (Abcam, ab1218) were used at 1:50 dilution. Secondary antibodies (including donkey anti-rabbit, anti-goat, or anti-mouse) each with different Alexa Fluor conjugations were all purchased from Life Technologies, and used at 1:200 dilution.

### Inertial microfluidic separation

EGFP^+^ cells purified by cell sorter were separated into two groups according to cell sizes using a high-throughput passive particle sorting technique based on inertial microfluidic principle^28^,^29^,^30^. Size separation of the EGFP^+^ cells was performed with a spiral micro-channel device fabricated with polydimethylsiloxane (PDMS). The device consisted of an 8-loop spiral microchannel with one inlet and two outlets. The radius of the spiral loops increases from 8 mm to 24 mm. The cross-section of the spiral micro-channel was trapezoidal with 600 μm in width and 80/130 μm in inner/outer heights, respectively^34^,^35^. The EGFP^+^ cells were suspended in 3 ml sterile PBS and loaded in a 10 ml syringe (Thermo Scientific, Japan), then pumped into the device inlet at a fixed flow rate of 1.7 ml/min using a syringe pump (PHD 2000, Harvard Apparatus, USA). The large and small EGFP^+^ cells were collected from the inner and outer outlets of the device, respectively.

### RNA sequencing and analysis

EGFP^+^ cells freshly isolated from mouse lung (D0) or from the colonies cultured at days 4 (D4) and 7 (D7) were used to extract RNA, 1~5 ng of total RNA was used to amplify transcripts using REPLI-g WTA Single Cell Kit (Qiagen). The amplification products were sequenced using NGS Ilumina Hiseq 2000 sequencing system (AITbiotech). Paired sequences were aligned with the mouse genome (version mm10) using Tophat2^36^. Raw counts of each genes of each sample were calculated by HTseq^37^. Differentially expressed genes between samples of D4 against D0, D7 against D0 and D7 against D4 were performed using the program edgeR at P-value < 0.05^38^. The gene expression level across different samples was normalized and quantified using the function of cpm. Differentially expressed genes were annotated using online functional enrichment analysis tool DAVID (http://david.ncifcrf.gov/)^39^. The enrichment analysis was performed separately for different groups of genes. We compared the differentially expressed genes with those published in the single cell RNA-seq data of fetal lung cells (E18)^32^. The fetal lung single cell data included expression profiles of bipotential progenitor cells (BP), AT1s, AT2s and club cells. The single cell data were normalized for each gene with mean equal to zero across all samples. To classify the samples of our *in vitro* cultured Club cells (D0, D4 and D7), the expression profiles were also normalized for each gene with mean equal to zero. Expressed genes from both data sets were identified if they are not zero in at least one single cell or one sample. Expression profiles of matched genes of two datasets were hierarchically clustered with uncentered Pearson method in MeV^40^. Both differentially expressed genes and signature genes of four cell types identified by principle component analysis (PCA) (Treutlein *et al*. 2014) were further analyzed to show the differentiating status of Club cells. All heatmap figures were visualized with MeV.

## Additional Information

**How to cite this article**: Zheng, D. *et al*. Differentiation of Club Cells to Alveolar Epithelial Cells *In Vitro. Sci. Rep.* **7**, 41661; doi: 10.1038/srep41661 (2017).

**Publisher's note:** Springer Nature remains neutral with regard to jurisdictional claims in published maps and institutional affiliations.

## Supplementary Material

Supplementary Information

Supplementary Table 1

Supplementary Table 2

Supplementary Table 3

Supplementary Table 4
