# Supplementary material for: Differentiation of Club Cells to Alveolar Epithelial Cells In Vitro
Source: Sci Rep. 2017 Jan 27;7:41661. doi: 10.1038/srep41661 (PMC5269679; doi:10.1038/srep41661)

**Differentiation of Club Cells to Alveolar Epithelial Cells In Vitro**

Dahai Zheng, Boon-Seng Soh, Lu Yin, Guangan Hu, Qingfeng Chen, Hyungwon Choi, Jongyoon Han, Vincent T. K. Chow, Jianzhu Chen

**Supplementary legends**

**Supplementary Figure S1.** Representative DAPI (blue), EGFP (green) and p63 (red) staining of colonies formed by EGFP^+^ club cells at day 7. Combinations of different channels are shown as indicated. Arrows indicate cells positive for both EGFP and p63. Scale bars: 20 µm.

**Supplementary Figure S2.** Transcriptional analysis of EGFP^+^ cells in 3-D culture. RNA of EGFP^+^ cells that were freshly isolated from mouse lung at day 0 (D0), or isolated from the cultured colonies at day 4 (D4) and day 7 (D7) were processed for RNA sequencing. Differentially expressed genes among the samples were identified. Shown are the expression levels of these genes in different samples (a) and the over lapping and non-over lapping of the differentially expressed genes among different samples (b).

**Supplementary Table 1.** The raw counts of different samples.

**Supplementary Table 2.** Lists of differentially expressed genes among different samples.

**Supplementary Table 3.** Functional enrichment of the differentially expressed genes among the samples.

**Supplementary Table 4.** List of genes identified from different samples by Principle Component Analysis (PCA) to classify the four published fetal lung cell types.

Supplementary Figure S1


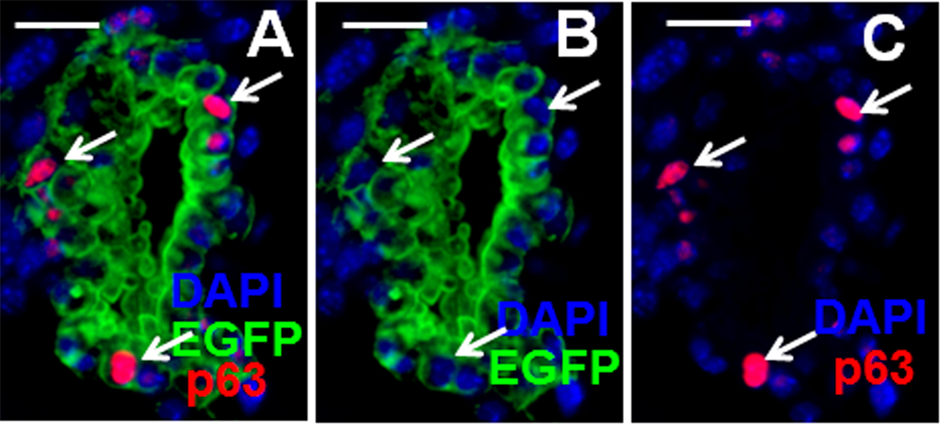


Supplementary Figure S2


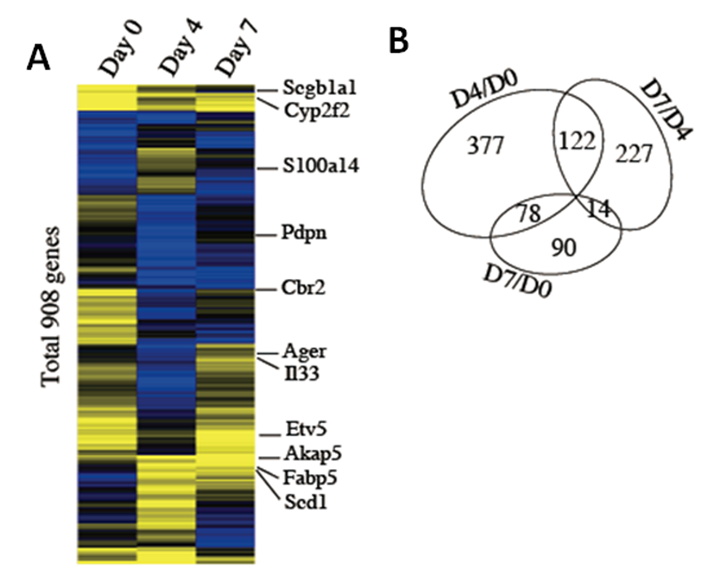

Supplement: Supplementary Information [file srep41661-s1.doc]
